# Slovenian midwifery students and their self-estimation of mindfulness: A cross-sectional study with modified MAAS

**DOI:** 10.18332/ejm/185649

**Published:** 2024-04-22

**Authors:** Laura Turin, Polona A. Mivšek

**Affiliations:** 1 Midwifery Department, Faculty of Health Sciences, University of Ljubljana, Ljubljana, Slovenia

**Keywords:** mindful awareness, midwifery students, stress management

## Abstract

**INTRODUCTION:**

We investigate the level of mindfulness among midwifery students, as mindfulness can have a major impact on their perception of stress and can increase the quality of their work after graduation.

**METHODS:**

A causal, non-experimental method of a cross-sectional study was used. We collected data using an online questionnaire that included a valid modified Mindful Attention Awareness Scale - MAAS. The sample consisted of Slovenian midwifery students in academic year 2022–2023.

**RESULTS:**

Fifty-five Slovenian midwifery students (82% response rate) participated in the study. The average score of all midwifery students on the 5-Likert modified MAAS was 3.2, with the lowest average score among second-year students. Those students who practiced mindfulness techniques in their free time had higher average scores than those who did not.

**CONCLUSIONS:**

The average MAAS score of our midwifery students was lower than in other foreign studies among nursing students. The study program should promote student mindfulness. This would benefit the graduates also later, when employed, as investing in midwives' well-being improves both their job satisfaction and women's experience of care. The Slovenian curriculum is often perceived as overwhelming by students. This might be the reason for their low mindfulness scores. Further analysis of the curriculum is needed to find solutions on how to integrate mindfulness techniques into the undergraduate curriculum.

## INTRODUCTION

Stress and burnout are common problems for midwives and midwifery students, as midwifery is very demanding emotional work^1^. Supporting women who are in pain and confronting various situations where a quick response is needed (e.g. postpartum hemorrhage or other complications) can be a great psychological burden for midwives. It is said that mindfulness can reduce stress and, at the same time, prevent burnout while improving the individual’s ability to concentrate and well-being^2^.

Incorporating mindfulness techniques into healthcare professionals’ learning programs, where they can learn to focus on breathing or a similar technique, focus on their inner world rather than the chaos around them, and shift attention from stressors to themselves, in the sense of being mindful and regulating their own emotions, can help healthcare professionals stay calm and deal effectively with the demanding situations at work^3^. Various techniques such as yoga, meditation, and learning different breathing techniques have been used in studies with healthcare professionals^4-7^. However, most studies have focused on the highly stressful work environments of nurses, while few have investigated the use of mindfulness techniques in midwives^8-9^.

One study from the UK found that 36.7% of midwives experience high levels of stress at work, and 67% of them report symptoms of burnout^9^. This study investigated how an 8-week mindfulness program can reduce stress levels. After the intervention, participants reported that they were more in control of themselves and felt less stress. At the same time, they rated the quality of their relationships higher, which can improve their satisfaction with working environment^1^. When conducting a similar intervention among nurses and nursing students, Guillaumie et al.^2^ found lower anxiety levels and better mental health, and participants reported inner peace and improved self-perception. In a study by Martín-Asuero and García-Banda^10^, the authors reported 35% less stress in healthcare workers who practiced mindfulness.

Healthcare students desired to learn mindfulness during undergraduate study^4,11^. In a British study, 5-week workshops were organized for medical students, who reported less stress and better concentration skills after the intervention^11^. Long-term effects reported in studies include better sleep, less anger, improved well-being^5,11^, better emotion regulation, and improved memory^6^. The benefits of mindfulness training were also found in an Australian study of medical students. The results confirmed a strong correlation between mindfulness and self-care. The authors concluded that the mental well-being of medical students should be a priority for educational institutions^12^. The study by Maher^13^ confirmed a negative correlation between the scores obtained on the Mindful Attention Awareness Scale (MAAS) and Perceived Stress Scale (PSS). Similar results were also found in studies on other health professionals^12,14^. Since mindfulness was rarely researched among midwifery students, we have decided to evaluate this in the only Slovenian faculty that educated midwives.

The midwifery program is offered via direct entry, lasts three years (180 ECTS), and is in line with the European directive for regulated professions. Students are educated to become assertive, independent healthcare professionals with high responsibility and a strong moral attitude. To practice safe, evidence-based, women-centered care, they should be attentive and mindful. Therefore, our study aimed to determine midwifery students’ self-assessment of mindfulness.

## METHODS

### Study design, setting, and participants

A quantitative approach was used; a cross-sectional study with a valid questionnaire to assess mindfulness – the modified MAAS initially developed by Brown and Ryan^15^ – was conducted. A purposive sample was used. The online survey was distributed to all 1st, 2nd, and 3rd year midwifery students at the only midwifery school in Slovenia in 2022 (n=67). Only fully completed questionnaires were analyzed (n=55); the response rate was 82%. Ethical approval was obtained and granted to ensure the integrity of the participants (13/10/22). In the questionnaire, the students first ticked the consent box to participate in the study. The aim of the study was explained to them, and they were informed that participation was voluntary and that they would be assured confidentiality.

### The MAAS for mindfulness

The MAAS^15^ consists of 15 statements that measure agreement on a Likert scale. The average agreement with the statements reflects the person’s mindfulness (a higher number indicates a more mindful attitude). The Slovenian version of the scale was prepared in a double-blind translation process; the scale was translated from English into Slovenian and back by two independent translators. The original scale and its translation were then checked in order to discuss potential content inconsistencies. None was found. From the 6-point Likert scale, we created a modified 5-point scale, merging categories 3 – ‘somewhat frequently’ and 4 – ‘somewhat infrequently’, similar to the study by Peterson^8^, in order to avoid a dispersion of responses. Because of the small sample and the fact that semantically, the difference between categories 3 and 4 is almost non-existent, we considered that all non-defined participants would choose 3 instead of spreading answers between categories 3 and 4.

The online questionnaire was created with the program 1KA^16^. The only demographic question added was the question about the year of study. As few midwifery students were male, we did not ask about gender. The research instrument was pilot-tested for validity on a sample of students in the additional year (before graduation) (n=11). The statements were found to be simple and straightforward, Cronbach’s alpha as a measure of internal consistency was calculated using SPSS 27.0^17^ and reached 0.96 (results of 0.58 to 0.97 are considered satisfactory)^18^.

The analysis was conducted using the SPSS program^17^. Basic descriptive measures were calculated (frequencies, mean values and ANOVA for testing statistically significant differences in students’ answers among study year groups).

## RESULTS

The study involved 19 out of 30 first-year midwifery students (63%), all 24 second-year students (100%), and 12 out of 13 third-year students (92%). They were all females aged 18–22 years.

Table 1 shows the frequency of agreement for the 15 statements on the modified MAAS and the average scores for each statement. Table 2 shows the average scores for the 15 statements on the modified MAAS by year of study.

**Table 1:** Agreement of midwifery students with statements, on the modified MAAS, Slovenia (N=55)

*Statements*		*Agreement*					
		*1=Almost always n (%)*	*2=Very frequently n (%)*	*3=Sometimes n (%)*	*4=Very infrequently n (%)*	*5=Almost never n (%)*	*Average score*
1	I could be experiencing some emotion and not be conscious of it until some time later.	5 (9)	13 (24)	30 (55)	5 (9)	2 (4)	2.7
2	I break or spill things because of carelessness, not paying attention, or thinking of something else.	2 (4)	1 (2)	11 (20)	20 (36)	21 (38)	4.0
3	I find it difficult to stay focused on what’s happening in the present.	3 (5)	5 (9)	21 (38)	20 (36)	6 (11)	3.4
4	I tend to walk quickly to get where I’m going without paying attention to what I experience along the way.	7 (13)	12 (22)	21 (38)	10 (18)	5 (9)	2.9
5	I tend not to notice feelings of physical tension or discomfort until they really grab my attention.	3 (5)	6 (11)	25 (45)	14 (25)	7 (13)	3.3
6	I forget a person’s name almost as soon as I’ve been told it for the first time.	13 (24)	14 (25)	16 (29)	6 (11)	6 (11)	2.6
7	It seems I am ‘running on automatic’, without much awareness of what I’m doing.	2 (4)	9 (16)	23 (42)	16 (29)	5 (9)	3.2
8	I rush through activities without being really attentive to them.	3 (5)	9 (16)	25 (45)	13 (24)	5 (9)	3.1
9	I get so focused on the goal I want to achieve that I lose touch with what I’m doing right now to get there.	0 (0)	13 (24)	18 (33)	18 (33)	6 (11)	3.3
10	I do jobs or tasks automatically, without being aware of what I‘m doing.	0 (0)	12 (22)	15 (27)	21 (38)	7 (13)	3.4
11	I find myself listening to someone with one ear, doing something else at the same time.	4 (7)	12 (22)	26 (47)	11 (20)	2 (4)	2.9
12	I drive places on ‘automatic pilot’ and then wonder why I went there.	2 (4)	2 (4)	17 (31)	14 (25)	20 (36)	3.9
13	I find myself preoccupied with the future or the past.	12 (22)	20 (36)	17 (31)	5 (9)	1 (2)	2.3
14	I find myself doing things without paying attention.	3 (5)	6 (11)	26 (47)	16 (29)	4 (7)	3.2
15	I snack without being aware that I’m eating.	5 (9)	7 (13)	16 (29)	10 (18)	17 (31)	3.5
**Overall average score**							**3.2**

**Table 2:** Agreement of midwifery students with statements, on the modified MAAS, according to the year of study, Slovenia (N=55)

*Statements*		*Average score*		
		*1st year (N=19)*	*2nd year (N=24)*	*3 rd year (N=12)*
1	I could be experiencing some emotion and not be conscious of it until some time later.	2.6	2.6	3.3
2	I break or spill things because of carelessness, not paying attention, or thinking of something else.	4.4	3.8	4.1
3	I find it difficult to stay focused on what’s happening in the present.	3.5	3.1	3.8
4	I tend to walk quickly to get where I’m going without paying attention to what I experience along the way.	2.9	2.7	3.2
5	I tend not to notice feelings of physical tension or discomfort until they really grab my attention.	3.4	3.0	3.7
6	I forget a person’s name almost as soon as I’ve been told it for the first time.	2.5	2.8	2.3
7	It seems I am ‘running on automatic’, without much awareness of what I’m doing.	3.5	2.8	3.7
8	I rush through activities without being really attentive to them.	3.0	3.0	3.8
9	I get so focused on the goal I want to achieve that I lose touch with what I’m doing right now to get there.	3.5	3.1	3.5
10	I do jobs or tasks automatically, without being aware of what I‘m doing.	3.5	3.1	3.9
11	I find myself listening to someone with one ear, doing something else at the same time.	2.6	3.0	3.3
12	I drive places on ‘automatic pilot’ and then wonder why I went there.	4.2	3.5	4.3
13	I find myself preoccupied with the future or the past.	2.3	2.2	2.6
14	I find myself doing things without paying attention.	3.1	3.0	3.8
15	I snack without being aware that I’m eating.	3.2	3.4	4.1
**Overall average score**		**3.2**	**2.8**	**3.5**

Modified MAAS: 1=almost always, 2=very frequently, 3=sometimes, 4=very infrequently, 5=almost never.

We can see that the lowest average score was for the statement: ‘I forget a person’s name almost immediately after it is first said to me’, which could mean that they often do not pay attention to conversations. However, the average score was still 2.6, and the highest score was 5, so we cannot consider this very low. The statement: ‘I break or spill things because I am careless, not paying attention or thinking about something else’ (average score 4=very infrequently), achieved the highest level of disagreement (74% of responses ‘almost never’ or ‘very infrequently’), which means that this is the less frequent situation they experience. This is somehow in contrast with frequent approval (58% ‘almost always’ or ‘very frequently’) with the statement: ‘I am preoccupied with the future or the past’, which can indicate that participants are not focused and attentive to the moment.

A comparison of the assessments between the students in the different years of study (Table 2) shows that, unlike in the first and third years of study, the second-year students had a lower average score of 2.2 for the statement: ‘I am preoccupied with the future or the past’, which means that they are often not present in the current situation. They were also the least mindful overall (overall average score 2.8).

Students in their 3rd year of midwifery training were the most mindful. The highest average score in their group was achieved by the statement: ‘I drive to places on ‘autopilot’ and then ask myself why I went there’ (average score 4.3), which means that they rarely experience such situations. In the group of first-year students, the statement: ‘I break or spill things because I am careless, not paying attention or thinking about something else’, achieved the highest average score (score 4.4), meaning this almost never happens to them. In the group of 2nd year midwifery students, none of the statements achieved an average score >4, indicating lower mindfulness than in the other two groups. Despite the differences in averages, one way ANOVA did not show any statistically significant differences in the students’ responses when compared by year of study (p>0.05).

We calculated the average MAAS scores when categorizing students in terms of previous training and practice of mindfulness techniques (some of them admitted to practice mindfulness). Those students who practiced mindfulness in their free time scored an average of 3.5 on the MAAS, while those who did not, scored an average of 3.1; the difference is not statistically significant (p>0.05).

## DISCUSSION

The study’s main aim was to determine whether midwifery students are mindful. The more points participants score on a MAAS, the more mindful they are^15^. The average on the modified MAAS for students in all three study cohorts was 3.2 (out of 5); we can conclude that they are mindful, although we expected higher scores. Since they will be healthcare practitioners with highly demanding and responsible work, we wish they would be very attentive. However, when comparing the overall results by year of study, third-year students did achieve a higher average score (3.5 out of 5).

We know that midwifery is very demanding and that a lack of presence in certain situations can put midwifery clients at risk. Inattentive midwifery can lead to professional errors and also affect midwife-client behavior. Not being mindful can reduce empathy and attention to subtle features of a trusting relationship with women^9^. Therefore, midwifery students should learn mindfulness during their studies^4,11^, to be able to practice it as graduates. In the original study by Brown and Ryan^15^, who developed the MAAS, the average score of the psychology students was 3.85, but we must be aware that they conducted the study on a 6-point Likert scale. Therefore, a study by Peterson^8^ who examined mindfulness among nursing students using a 5-point Likert scale, is more comparable to our study. They achieved an average score of 4.1 in their pre-mindfulness intervention test.

Since our results are lower (score of 3.2), we question whether study programs could promote (or discourage) mindfulness in students. Our students criticized in their annual evaluations the organization of their studies, claiming that studying midwifery involves too many commitments; they do clinical practice, attend lectures and seminars at the faculty or study – they are constantly engaged, and they have many tasks at once. This could be the reason why they cannot concentrate and focus.

As mentioned, analysis of the curriculum and student reports have shown that students are overwhelmed with their commitments. The program of study, which lasts three years and is designed to give students all theoretical and practical competencies for the job, is very dense and complex. This could explain the lower scores of students on the MAAS; they frantically follow all the prescribed activities and cannot reflect on their study process in depth. Particularly low scores were found in the second year of study (score of 2.8), which is probably because they have to take on more responsibility and at the same time realize that they only have one year left to achieve all the expected study goals.

In response to this analysis, the midwifery teaching team has already sent a four-year study program to accreditation, allowing for practical and theoretical semesters, more time for in-depth study, and a more deliberate approach to the study process. More time is also devoted to the individual assessment of students’ professional development.

Similar results to those in our study were also confirmed by Dubert et al.^6^ among nursing students; students achieved the highest levels of mindfulness in the first year of study and then less and less after that. They explained this by the fact that individuals become more aware of professional demands as their studies progress and that these demands cause stress^6^. As nursing and midwifery are regulated professions in the EU, this problem of student overload could be a common problem across all EU universities with these two-degree programs. However, few studies on mindfulness among midwifery students have been conducted so far, and our study was the first study among Slovenian midwifery students. Further studies on mindfulness in different EU countries could illuminate how to design a midwifery curriculum that fulfils EU regulatory requirements while enabling a student-friendly environment with the opportunities for mindful learning.

Midwifery teachers should strive to create a teaching environment that supports mindfulness. Maher^13^ writes that 82% of participants in her study were interested in learning techniques that promote mindfulness during their studies. The benefits of including mindfulness techniques in the study program have already been confirmed^4,11^. In our study, the average scores were also slightly better in students who practiced mindfulness-promoting techniques (especially yoga). Similarly, in a study by Brown and Ryan^15^, the participants practicing mindfulness techniques achieved an average score of 4.38 (while others achieved a score of 3.85), and in a study by Erkin and Senuzun^7^ among nursing students, the group practicing yoga achieved a 0.4 higher average score. Including skills that would help students alleviate stress in the basic study program might also help them later in life.

### Limitations

Our study has some limitations. A limitation is the small sample size, although we did include all Slovenian students studying midwifery in 2022. Nevertheless, the small sample limits the generalization of the findings. We used a 5-point Likert scale because we did not want the answers to scatter too much; this way, all undefined participants could choose the value three instead of 3 or 4. As we had a small sample, we believe this was a solution for not dispersing answers into many categories. However, it was more challenging to find other studies to compare the results with, but some authors^8^ came to the same conclusion. An essential limitation of the study is that some participants already use or have used mindfulness techniques in their free time, which could affect (and falsely improve) their MAAS scores. We could also use factor analysis for construct validity to improve the process of validating the scale.

In the future, a qualitative study could provide more detailed information about students’ perceptions of barriers to mindfulness. A curriculum analysis should also be conducted to determine how mindfulness techniques could be integrated into the basic midwifery study program.

## CONCLUSIONS

Including subjects in the undergraduate curriculum that promote mindfulness in midwifery students could be beneficial. The literature review found that mindfulness is important for all healthcare professionals, not just midwives. Further research is needed to determine which techniques would be most appropriate. If students were to learn these methods during their studies, they could also apply them on the job – midwifery is a demanding and stressful profession that often causes midwives to burn out. These strategies could also prevent the negative effects of the working environment on clinical midwives in the long-term.

## Data Availability

The data supporting this research cannot be made available for privacy or other reasons.
